# Vasohibin 2 promotes malignant behaviors of pancreatic cancer cells by inducing epithelial‐mesenchymal transition via Hedgehog signaling pathway

**DOI:** 10.1002/cam4.1752

**Published:** 2018-10-14

**Authors:** Ye Zhang, Xiaofeng Xue, Xiaoqian Zhao, Lei Qin, Yu Shen, Huiqiang Dou, Jialin Sun, Tong Wang, Da‐Qing Yang

**Affiliations:** ^1^ Wuxi People's Hospital Nanjing Medical University Wuxi China; ^2^ The Hormel Institute University of Minnesota Austin Minnesota; ^3^ College of Clinical Medicine Nanjing Medical University Nanjing China; ^4^ First Affiliated Hospital of Suzhou University Suzhou University Suzhou China; ^5^ The Masonic Cancer Center University of Minnesota Austin Minnesota

**Keywords:** epithelial‐mesenchymal transition, Hedgehog signaling, pancreatic cancer, VASH2, ZEB1/2

## Abstract

**Background:**

Based on previous findings, we hypothesized that Vasohibin 2 (VASH2) protein may induce epithelial‐mesenchymal transition (EMT) of pancreatic cancer (PC) cells by promoting the malignant behaviors of these cells. The present study aimed to test this hypothesis and explore the possible mechanisms involved.

**Methods:**

The expression of VASH2 in PC tissues and cell lines was detected by quantitative real‐time PCR and Western blot. PC cells with overexpression or knockdown of VASH2 were used to examine the involvement of VASH2 in EMT by detecting the expression of epithelial (E‐cadherin) and mesenchymal (vimentin) markers and EMT‐related transcription factor ZEB1/2, in gemcitabine resistance and tumor cell invasion by apoptosis and invasion assays, and in cancer stem cell‐like phenotypes by detecting the proportion of CD24^+^CD44^+^ and side population (SP) cells in PC cells with flow cytometry. The impact of VASH2 overexpression and knockdown on components of the Hedgehog signaling pathway was also assessed.

**Results:**

We found that VASH2 was highly expressed in PC tissues and cells. It promoted the EMT of PC cells by altering ZEB1/2 expression. VASH2 also stimulated invasion and chemotherapeutic resistance of PC cells and increased the proportion of cancer stem‐like cells in PC cells. VASH2 did so by upregulating the expression of multiple molecules in the Hedgehog signaling pathway of PC cells.

**Conclusion:**

VASH2 promotes malignant behaviors of PC cells by inducing EMT *via* activation of the Hedgehog signaling pathway.

## 1 INTRODUCTION

Pancreatic cancer (PC) is the fourth leading cause of cancer death accounting for nearly 7% of all deaths in cancer patients.^1^ Although many therapeutic modalities such as surgery and chemotherapeutics, either alone or in combination, have been used for the treatment of PC, there has been no significant improvement in the death rates of PC from 1930 to 2011, mainly due to its malignant behaviors including rapid progression, aggressive invasion, early metastasis, and drug resistance.^2^, ^3^ Many studies have been carried out to explore the unique characteristics of this malignancy, but it remains largely unclear how PC acquires these malignant behaviors. Thus, there is an urgent need to develop alternative therapeutic strategies that target the pathways responsible for the malignant behaviors of PC.

Epithelial‐mesenchymal transition (EMT), a transient and reversible process in which polarized epithelial cells acquire a fibroblastoid or mesenchymal phenotype, is associated with the high motility, invasiveness, metastasis, and drug resistance of tumor cells.^4^, ^5^, ^6^ Cells that have undergone EMT exhibit cancer stem cell‐like features and are able to invade across basement membranes and stromal tissues.^7^ Our previous study has shown that EMT correlates with cancer stem cell‐like phenotypes, mainly increased proportion of CD24^+^CD44^+^ and CD133^+^ cells in PC.^8^ Thus, clarifying the mechanism of EMT in PC may help to develop effective targeted treatments for PC.

Vasohibin 2 (VASH2), first described by Shibuya *et a l,* 9 belongs to the VASH family, which includes VASH1 and VASH2. Although VASH2 was initially discovered as an angiogenesis inhibitor,^9^ later results indicated that VASH2 in fact promotes angiogenesis during injury repair.^10^ Our results also demonstrated that VASH2 promotes tumor angiogenesis in hepatocellular carcinoma (HCC) cells.^11^ Subsequently, VASH2 was discovered as a promoter of EMT in human malignancies.^12^ VASH2 is localized in both cytosol and nucleus and is abnormally expressed in various types of cancer cells. In PC, overexpression of VASH2 is associated with accelerated tumor malignant transformation and reduced chemosensitivity to gemcitabine.^13^ Our study also suggests that VASH2 is involved in promoting EMT of HCC cells.^14^ Therefore, we hypothesized that VASH2 may regulate EMT in PC cells.

To test this hypothesis, we examined the role of VASH2 in the EMT and various malignant behaviors, including gemcitabine resistance, invasion, and increased cancer stem cell‐like phenotypes, of PC cells. We also assessed the impact of VASH2 on the expression of key molecules of the Hedgehog signaling pathway. It is expected that our findings will provide a theoretical basis for developing new targeted therapies for PC.

## 2 MATERIALS AND METHODS

### 2.1 PC cell lines

The human pancreatic cancer cell lines PANC‐1, SW1990, CFPAC, HPAC, Capan‐1, Miapaca‐2, and BxPc‐3 from ATCC were used in the study. HPAC cells were cultured in DMEM/F12 (Hyclone, Logan, Utah, USA), CFPAC cells were cultured in IMDM (Hyclone), and other cell lines in DMEM (Hyclone), supplemented with 10% fetal bovine serum (FBS; Gibco, Waltham, MA, USA), 100 mg/mL penicillin and 100 mg/mL streptomycin at 37°C in a humidified chamber supplemented with 5% CO_2_.

### 2.2 Western blot

Western blotting was performed using standard protocols. Briefly, cells were sonicated in RIPA buffer, and 30 μg proteins was resolved on 12% polyacrylamide SDS gels and transferred to polyvinylidene difluoride (PVDF) membranes. The membranes were blocked with 5% BSA (bovine serum albumin) in TBS (tris‐buffered saline), incubated overnight at 4°C with the appropriate primary antibodies. The antibodies used included those against VASH2 (ab116640, Abcam, Cambridge, UK), GAPDH (AG019‐1, Beyotime, Shanghai, China), Vimentin (ab8069, Abcam), E‐cadherin (ab11512, Abcam), ZEB1 (ab124512, Abcam), ZEB2 (ab25837, Abcam), Bcl‐2 (ab32124, Abcam), SMO (ab38686,, Abcam), Gli‐1 (ab49314, Abcam), and Gli‐2 (ab167389, Abcam).

### 2.3 Plasmid construction and lentivirus packaging

The construction of plasmids for knockdown and overexpression of VASH2 (PANC‐1‐shVASH2, PANC‐1‐shcont, BxPc‐3‐VASH2, and BxPc‐3‐con) and lentivirus packaging were performed as described previously.^14^ PANC‐1 and BxPc‐3 cell lines were used to knock down and overexpress VASH2, respectively.

### 2.4 Morphology and immunofluorescence

Cells were plated into chamber slides (Falcon, Edison, NJ, USA) and fixed with 3% formaldehyde. Samples were then blocked with 5% BSA and incubated with anti‐E‐cadherin, anti‐VASH2 and anti‐Vimentin overnight at 4˚C. Rabbit IgGs were used as a negative control. After incubating with the primary antibodies, cells were washed three times in PBS (phosphate‐buffered saline) followed by 60 minutes of incubation at room temperature with anti‐rabbit FITC (fluorescein isothiocyanate) and secondary antibody. After washing with PBS, the cells were mounted using FLUORO‐GEL II with DAPI (4′,6‐diamidino‐2‐phenylindole) (Electron Microscopy Science, Pennsylvania). Fluorescence was analyzed using the fluorescence microscopy (Nikon, Japan).

### 2.5 MTT assay

PANC‐1 and BxPC‐3 cells were splited into 96 wells and cells were grown to 80%‐90% subconfluence. Gemcitabine was then added to the cells at the indicated concentrations. After 48 hours of treatment, MTT (Thiazolyl Blue Tetrazolium Bromide**)** was added into the medium and the cell culture mixture was incubated for another 4 hours. The cell culture medium was then mixed with 150 μL DMSO (dimethyl sulfoxide) for 10 minutes before the measurement of optical absorbance at 490 nm.

### 2.6 Detection of cell apoptosis

Apoptosis was determined using FITC Annexin V Apoptosis Detection Kit (Beyotime, Shanghai, China) following the manufacturer's instructions. Briefly, cells were centrifuged, washed in PBS, resuspended in 100 μL of 1 ×  binding buffer and stained with 5 μL of FITC (fluorescein isothiocyanate)‐labelled Annexin V and 5 μL of propidium iodine at room temperature for 15 minutes in the dark. The stained cells were then analyzed by flow cytometry.

### 2.7 In vitro invasion assay

The matrigel invasion assay was performed using 24‐well transwell chambers (8.0 μm pore size with polycarbonate membrane; Corning Life Sciences, Lowell, MA, USA) following the manufacturer's instructions. Briefly, the lower compartment was seeded with 5 × 10^4^ cells in 600 μL complete medium and incubated for 12 hours, and then the medium was changed to serum‐free medium. The upper chamber was coated with 100 μL of 1 mg/mL Matrigel (BD Biosciences, Bedford, MA) and incubated for 3 hours when 0.6 mL of 10% FBS‐DMEM was added to the lower chamber. After incubation for 24 hours, noninvading cells were removed with cotton swabs. Cells migrating to the bottom of the membrane were stained with 0.1% crystal violet for 30 minutesat 37ºC, washed with PBS and photographed.

### 2.8 Flow cytometry

Flow cytometry was performed using the standard protocol. Briefly, cells were labeled with 5 μg/mL anti‐CD44 (APC) and 2.5 μg/mL anti‐CD24 (PE) antibodies for 10 minutes at 37ºC, using 2.5 μg/mL rat IgG2b (APC) and 5 μg/mL mouse IgG1 (PE) as isotype controls, respectively. All antibodies were purchased from eBioscience (San Diego, CA). Flow cytometry was performed using a BD FACSCalibar flow cytometer. Analysis of side population (SP) cells was performed as described previously.^14^

### 2.9 Statistical analysis

All experiments were repeated in triplicate. Statistical significance was determined by Student's *t* test. *P*‐values < 0.05 were considered statistically significance.

## 3 RESULTS

### 3.1 Expression of VASH2 in human pancreatic ductal adenocarcinoma tissues and pancreatic cancer cell lines

**Figure 1:**
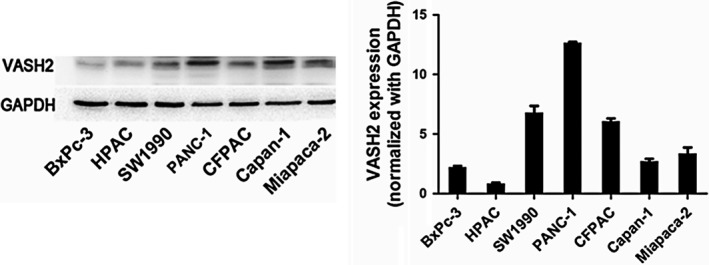
Expression of VASH2 in pancreatic cancer cell lines. Western blot analysis of VASH2 protein expression was performed in various pancreatic cancer cell lines, including Miapaca‐2, Capan‐1, CFPAC‐1, PANC‐1, SW1990, HPAC, and BxPc‐3

Our previous results have shown increased expression of VASH2 in pancreatic ductal adenocarcinoma tissues versus matched tumor‐adjacent benign tissues by immunohistochemistry staining.^13^ We have confirmed this finding using qRT‐PCR (data not shown). We further detected the expression of VASH2 protein in pancreatic cancer cell lines (Miapaca‐2, Capan‐1, CFPAC‐1, PANC‐1, SW1990, HPAC, and BxPc‐3) by Western blot, which revealed that PANC‐1 cells had higher expression of VASH2, while BxPc‐3 cells displayed lower VASH2 expression (Figure 1). Therefore, we selected the PANC‐1 cell line to knock down and the BxPc‐3 cell line to overexpress VASH2 in subsequent experiments.

### 3.2 PC cells overexpressing VASH2 exhibit morphologic and molecular changes consistent with EMT

**Figure 2:**
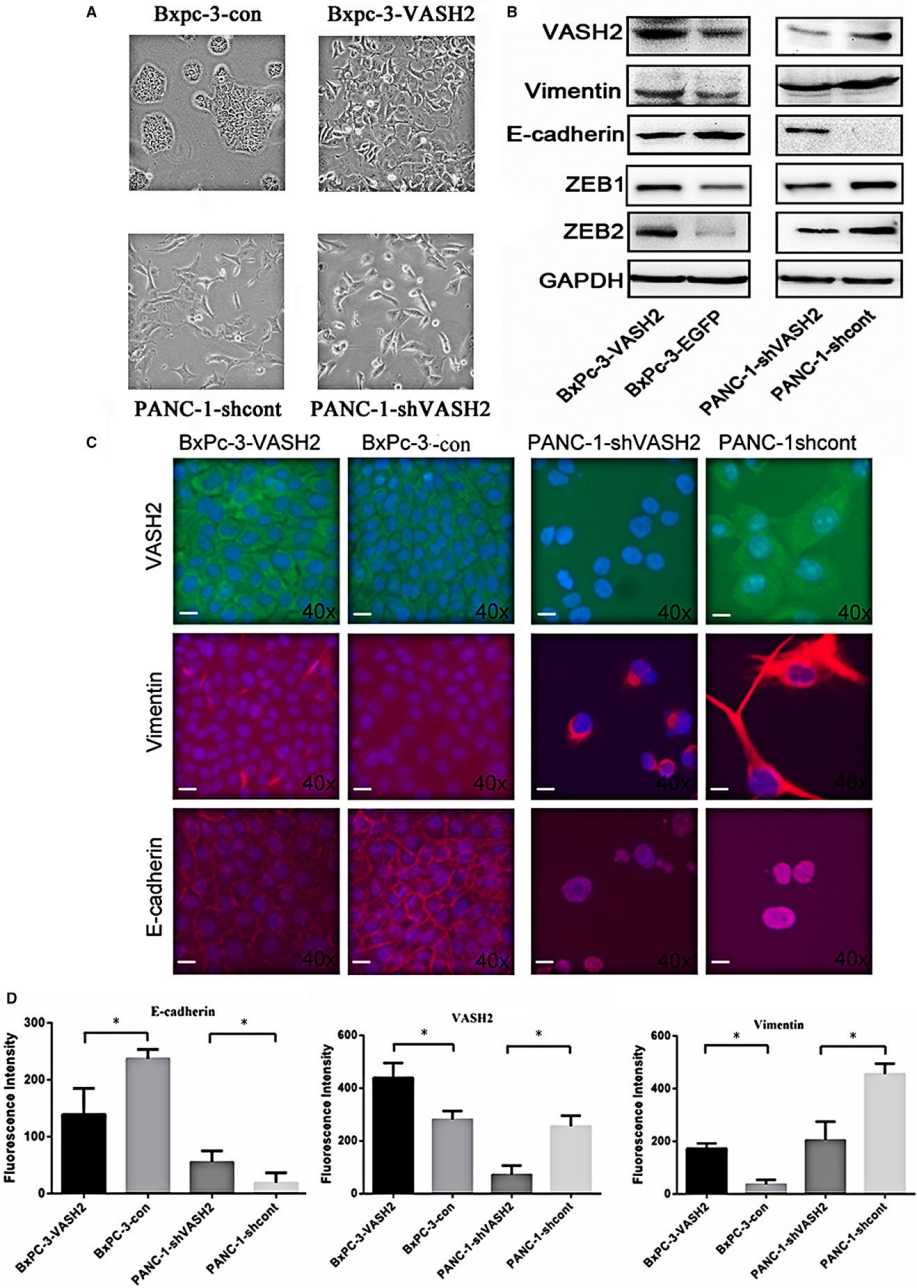
VASH2 expression is associated with morphologic changes and altered expression of EMT biomarkers consistent with EMT. A, Morphologic changes in VASH2‐overexpressing BxPc‐3 cells and PANC‐1 cells with VASH2 knockdown; B, Western blot analysis of E‐cadherin, vimentin, and ZEB1/2 in VASH2‐overexpressing BxPc‐3 cells and PANC‐1 cells with VASH2 knockdown. C and D, Immunofluorescence localization of VASH2, E‐cadherin and vimentin in VASH2‐overexpressing BxPc‐3 cells and PANC‐1 cells with VASH2 knockdown. The scale bar (20 μm) was at the lower left corner of each panel. The average signal densities ± SEM for the immunostained proteins were shown in D

As shown in Figure 2A, VASH2‐overexpressing BxPc‐3 cells exhibited an elongated irregular fibroblastoid morphology. In contrast, PANC‐1 cells with VASH2 knockdown had an epithelial‐like appearance and grew in clusters. These changes suggest that VASH2 may be associated with EMT in pancreatic cancer cells. To further confirm this finding, we determined the expression of markers of epithelial and mesenchymal phenotypes by Western blot and immunofluorescence, which showed that the expression of E‐cadherin, an epithelial marker, was reduced in VASH2‐overexpressing BxPc‐3 cells but increased in PANC‐1 cells with VASH2 knockdown, while vimentin, a mesenchymal marker, showed an opposite expression pattern (Figure 2B,C and D). Of note, the expression levels of ZEB1 and ZEB2,^14^ which were previously found to mediate the function of VASH2, were increased in VASH2‐overexpressing BxPc‐3 cells and reduced in PANC‐1 cells with VASH2 knockdown. Collectively, these results suggest that VASH2 may promote EMT in PC cells possibly by altering ZEB1/2 expression (Figure 2B).

### 3.3 VASH2 promotes gemcitabine resistance and invasion in PC cells

**Figure 3:**
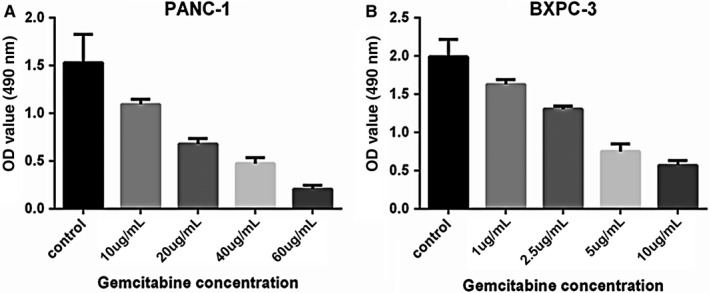
The effect of gemcitabine on cell growth of BxPC‐3 and PANC‐1 cells. Subconfluent PANC‐1 (A) and BxPC‐3 (B) cells were treated with gemcitabine at the indicated concentrations for 48 h, and the IC50 of PANC‐1 and BXPC‐3 for gemcitabine were determined to be 18.67 and 3.78 μg/mL, respectively

**Figure 4:**
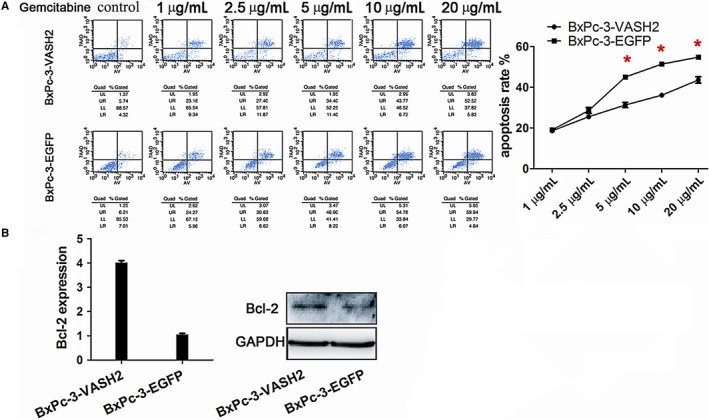
VASH2 promotes the gemcitabine resistance of BxPc‐3 cells by increasing their anti‐apoptotic ability via upregulating Bcl‐2. A, Flow cytometry analysis of apoptosis of VASH2‐overexpressing BxPc‐3 cells and control BxPc‐3 cells treated with gemcitabine at indicated doses (*P <* *0.05). B, Western blot analysis of Bcl‐2 expression in VASH2‐overexpressing BxPc‐3 cells and control BxPc‐3 cells

**Figure 5:**
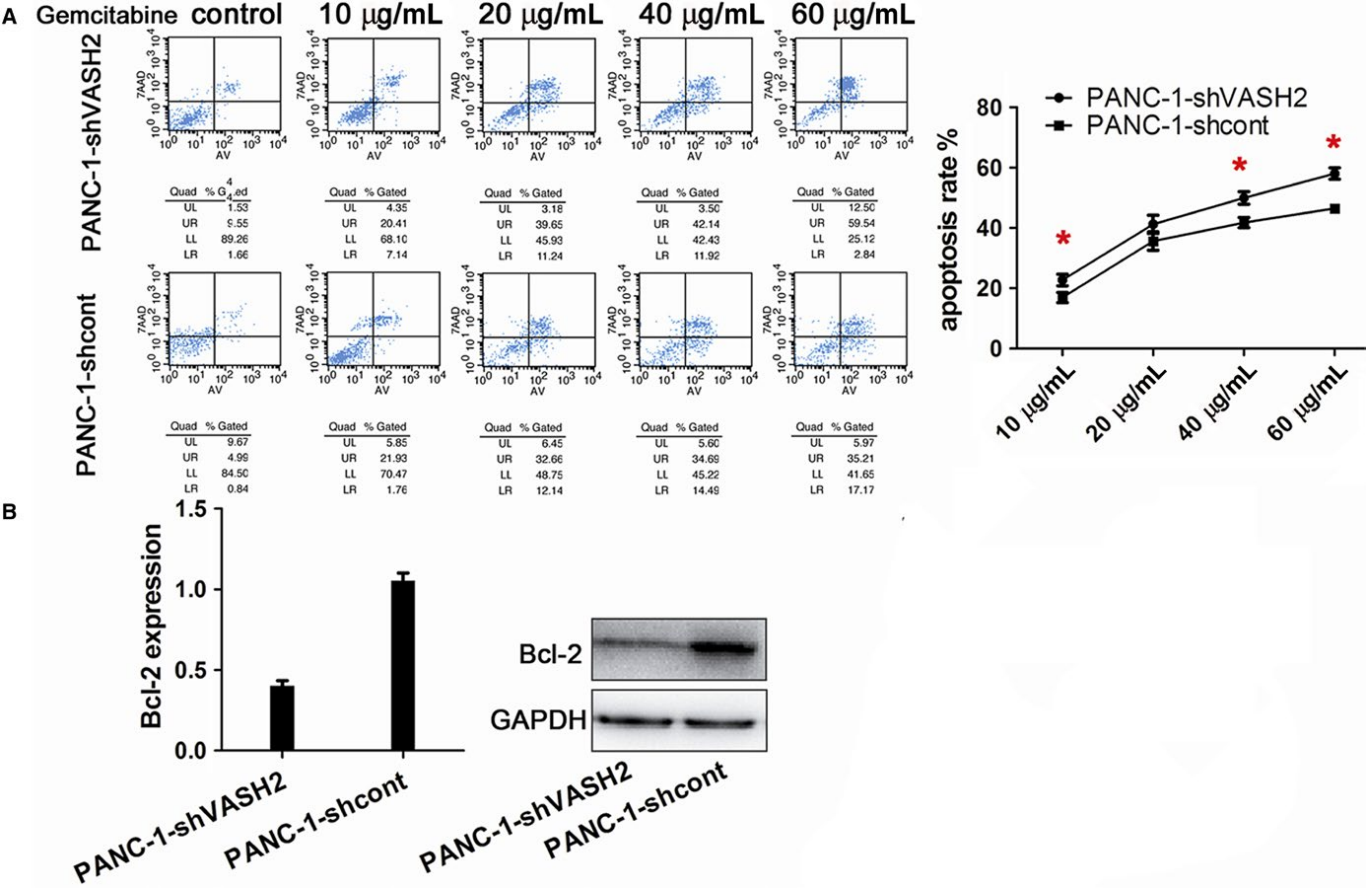
VASH2 promotes the gemcitabine resistance of PANC‐1 cells by increasing their antiapoptotic ability via upregulating Bcl‐2. A, Flow cytometry analysis of apoptosis of PANC‐1 cells with VASH2 knockdown and control PANC‐1 cells treated with gemcitabine at indicated doses (*P <* *0.05). B, Western blot analysis of Bcl‐2 expression in PANC‐1 cells with VASH2 knockdown and control PANC‐1 cells

It is known that EMT can promote gemcitabine resistance and invasion of tumor cells.^15^ After measuring the IC50 of gemcitabine in BxPC‐3 and PANC‐1 cells (Figure 3), we examined the impact of VASH2 expression on the apoptosis of PC cells after treating VASH2‐overexpressing BxPc‐3 cells and control BxPc‐3 cells with 1.0‐20 μg/mL gemcitabine, and PANC‐1 cells with VASH2 knockdown and control PANC‐1 cells with 10‐60 μg/mL gemcitabine, respectively. Flow cytometry analysis showed a significant increase in apoptotic cells in control BxPc‐3 cells compared to VASH2‐overexpressing BxPc‐3 cells treated with 5.0, 10 and 20 μg/mL gemcitabine (Figure 4A), and a significant increase in apoptotic cells in PANC‐1 cells with VASH2 knockdown compared to control PANC‐1 cells treated with 40 and 60 μg/mL gemcitabine (Figure 5A). Furthermore, the expression of the antiapoptotic gene Bcl‐2 was measured by Western blot, which showed that VASH2 overexpression increased Bcl‐2 expression while VASH2 knockdown decreased Bcl‐2 expression (Figure 4B and 5B). These findings suggest that VASH2 promotes the gemcitabine resistance of PC cells by increasing their antiapoptotic ability *via* upregulating Bcl‐2.

**Figure 6:**
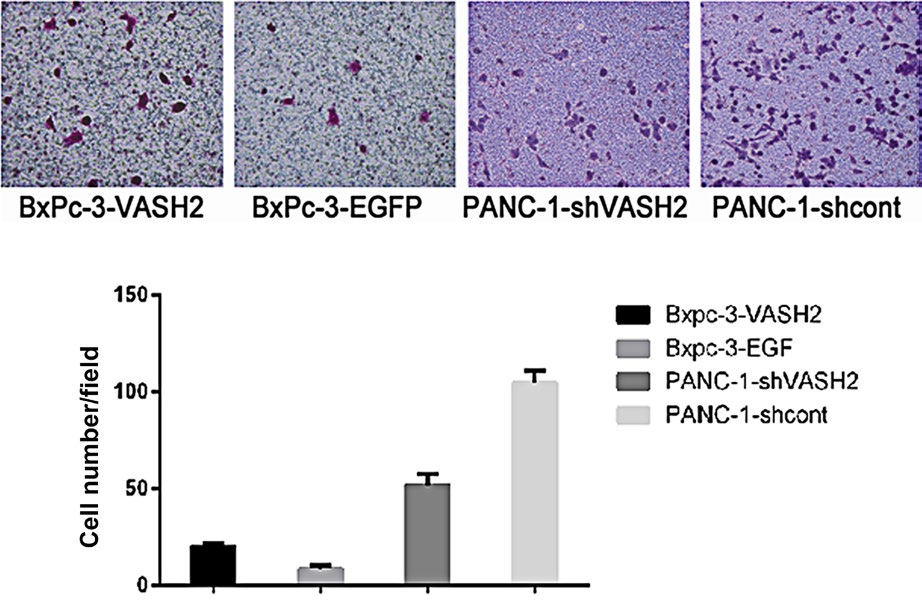
VASH2 promotes PC cell invasion in vitro. The invasive ability of VASH2‐overexpressing BxPc‐3 cells, control BxPc‐3 cells, PANC‐1 cells with VASH2 knockdown and control PANC‐1 cells was assessed by in vitro invasion assay and compared

To determine the impact of VASH2 expression on PC cell invasion, we evaluated the invasive ability of VASH2‐overexpressing BxPc‐3 cells and PANC‐1 cells with VASH2 knockdown by in vitro invasion assay. The results showed that the invasive ability of VASH2‐overexpressing BxPc‐3 cells significantly increased compared to control BxPc‐3 cells (Figure 6), while that of PANC‐1 cells with VASH2 knockdown significantly decreased compared to control PANC‐1 cells. Thus, these results suggest that VASH2 promotes PC cell invasion in vitro.

### 3.4 VASH2 increases the proportion of CD24^+^CD44^+^ cells and SP cells in PC cells

**Figure 7:**
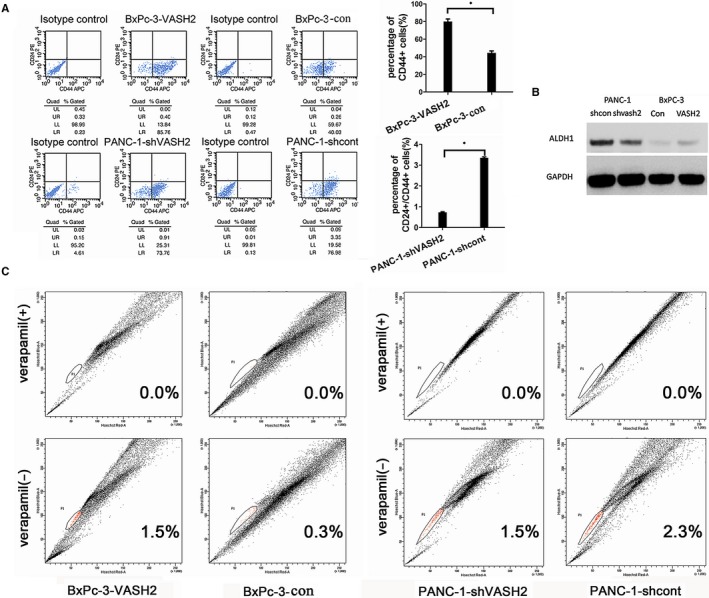
VASH2 expression increases the proportion of CD24^+^ CD44^+^ cells and SP cells in PC cells. A, The proportion of CD24^+^ CD44^+^ cells in VASH2‐overexpressing BxPc‐3 cells, PANC‐1 cells with VASH2 knockdown and control cells. B, The effect of VASH2 knockdown or overexpression on ALDH1 expression in PANC‐1 cells and BxPc‐3 cells, respectively. C, The proportion of SP cells, shown as P1 or population 1 in each panel, in VASH2‐overexpressing BxPc‐3 cells, PANC‐1 cells with VASH2 knockdown and control cells

It is well documented that cancer cells positive for the cell surface antigens CD24, and CD44 exhibit increased self‐renewal and tumor‐initiating potential. Furthermore, cells in a side population (SP) exhibit distinguishing stem cell‐like characteristics. Thus, the proportion of CD24^+^CD44^+^ cells and SP cells in cultured PC cells was detected by flow cytometry.^16^ As shown in Figure 7A, the proportion of CD24^+^CD44^+^ cells in PANC‐1 cells with VASH2 knockdown significantly decreased compared to mock‐transfected PANC‐1 cells (*P *<* *0.01). Interestingly, the proportion of CD44^+^ cells in VASH2‐overexpressing BxPc‐3 cells was significantly higher than that in mock‐transfected BxPc‐3 cells (*P *<* *0.01), whereas the proportion of CD24^+^ cells in VASH2‐overexpressing BxPc‐3 cells was similar to that in control cells. We also found that VASH2 knockdown caused reduced expression of aldehyde dehydrogenase 1 (ALDH1) in PANC‐1 cells and VASH2 overexpression led to increased expression of ALDH1, another well‐known marker of cancer stem cells (Figure 7B).

The proportion of SP cells in PC cells was also detected by flow cytometry (Figure 7C), which revealed that the proportion of SP cells in PANC‐1 cells with VASH2 knockdown was reduced compared to mock‐transfected PANC‐1 cells, and the proportion of SP cells in VASH2‐overexpressing BxPc‐3 cells was higher than that in mock‐transfected BxPc‐3 cells. Taken together, the above results suggest that VASH2 could influence the proportion of cancer stem‐like cells in PC cells.

### 3.5 VASH2 upregulates the expression of molecules of the Hedgehog signaling pathway

**Figure 8:**
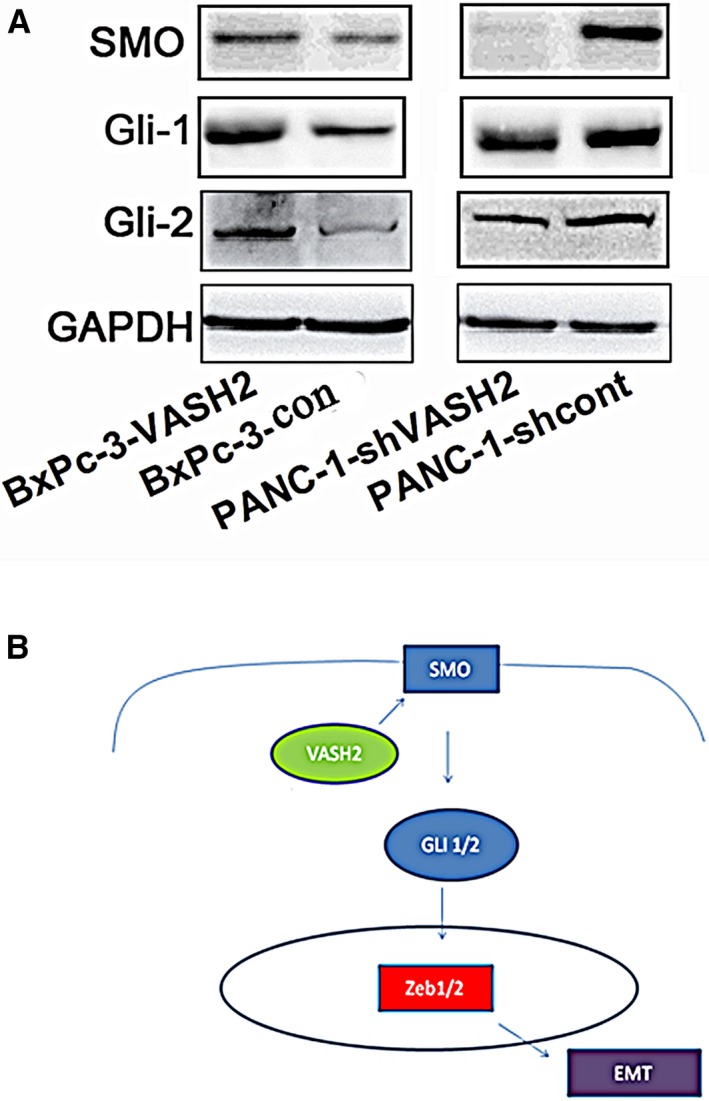
A, VASH2 regulates the expression of molecules of the Hedgehog signaling pathway in PC cells. The expression of SMO, Gli‐1, and Gli‐2 in VASH2‐overexpressing BxPc‐3 cells, PANC‐1 cells with VASH2 knockdown, and control cells was detected by Western blot. GAPDH was used as loading controls. B, A diagram illustrating the mechanism responsible for regulation of EMT by VASH2 in PC cells

Finally, we assessed the effect of VASH2 expression on the Hedgehog signaling pathway, which can alter the invasive and migratory capacity of PC cells and regulate EMT in PC,^17^ by detecting the expression of key molecules of this pathway by Western blot. We found that overexpression of VASH2 upregulated the expression of SMO, Gli‐1, and Gli‐2, while VASH2 knockdown downregulated the expression of these proteins (Figure 8A). These results suggest that VASH2 regulates the malignant behaviors of PC cells possibly *via* activation of the Hedgehog signaling pathway.

## 4 DISCUSSION

In the present study, we discovered that VASH2 expression is significantly increased in PC tissues and cell lines. Overexpression of VASH2 promotes EMT, cell invasion, and gemcitabine resistance and increases the proportion of stem‐like cells in PC cells by altering ZEB1/2 expression through upregulation of the Hedgehog signaling pathway.

Several studies have shown that VASH2 is highly expressed in HCC, breast cancer, and ovarian cancer, and that there is a close association between VASH2 expression and EMT in these malignancies.^13^, ^14^, ^18^ However, the role of VASH2 in the EMT process of PC cells remains unclear. In this study, we found that VASH2 expression is significantly elevated in PC tissues and VASH2 promotes EMT in PC cell lines, indicating that VASH2 may have a similar role in PC as in other tumors. Overexpression of VASH2 has also been demonstrated to accelerate malignant transformation and promote gemcitabine resistance in PC.^13^, ^19^ Our study has further shown that VASH2 may promote these malignant behaviors, including cell invasion and gemcitabine resistance, in PC cells by stimulating the EMT process in these cells.

Previous studies have found that EMT can enhance the invasive, migratory, and metastatic ability of PC cells,^8^ and these behaviors of PC cells were closely related with cancer stem cell‐like cell populations such as SP cells and CD24^+^CD44^+^ cells.^20^, ^21^ In agreement with this, we found that VASH2 increased the proportion of SP cells and CD24+ CD44+ cells in PC cells. Of note, the proportion of CD44+ cells in BxPc‐3 overexpressing VASH2 is significantly increased. As a receptor for extracellular matrix components, CD44 is closely linked to the metastasis of PC. It can also stimulate the EMT by activating two main proteins of the EMT pathways, Akt and NF‐kB.^22^, ^23^, ^24^ The finding that VASH2 can significantly increase the proportion of CD44+ cells suggest that VASH2 may promote the metastasis of PC by increasing the proportion of cancer stem cell‐like cells in PC cells.

Hedgehog signaling governs a wide variety of biological and molecular processes including tumorigenesis. Inhibition of Hedgehog signaling can suppress EMT, invasion, chemo‐resistance, stem‐like properties and metastasis of PC cells.^17^ Interestingly, overexpression of ZEB1/2 is also associated with these malignant behaviors of PC cells.^7^ Our findings that overexpression of VASH2 upregulates Hedgehog signaling, and knockdown of VASH2 downregulates Hedgehog signaling strongly suggest that VASH2 promotes malignant behaviors of PC cells via hedgehog signaling. Thus, it is conceivable that VASH2 may regulate the EMT process in PC cells by modulating the expression of ZEB1/2 through activation of the Hedgehog signaling pathway (Figure 8B).

In conclusion, our study demonstrates that VASH2 promotes the invasion and gemcitabine resistance as well as other malignant behaviors of PC cells by inducing EMT. VASH2 achieves this by activating the Hedgehog signaling pathway, leading to enhanced expression of ZEB1/2. Our study thereby validates VASH2 as a promising therapeutic target for PC.

## CONFLICT OF INTEREST

None declared.
